# Did you hear about HIV self-testing? HIV self-testing awareness after community-based HIVST distribution in rural Zimbabwe

**DOI:** 10.1186/s12879-022-07027-9

**Published:** 2022-01-13

**Authors:** Anke Rotsaert, Euphemia Sibanda, Karin Hatzold, Cheryl Johnson, Elizabeth Corbett, Melissa Neuman, Frances Cowan

**Affiliations:** 1 Department of Public Health, Institute of Tropical Medicine, Nationalestraat 155, 2000 Antwerp, Belgium; 2 Centre for Sexual Health and HIV AIDS Research (CeSHHAR), Harare, Zimbabwe; 3 Liverpool School of Tropical Medicine, Liverpool, UK; 4 Population Services International, Cape Town, South Africa; 5 Global HIV, Hepatitis and STI Programmes, World Health Organization, Geneva, Switzerland; 6 Department of Clinical Research and Infection Disease, London School of Hygiene and Tropical Medicine, London, UK; 7 Faculty of Epidemiology and Population Health, London School of Hygiene and Tropical Medicine, London, UK

**Keywords:** HIV self-testing, Community-based distribution

## Abstract

**Background:**

Several trials of community-based HIV self-testing (HIVST) provide evidence on the acceptability and feasibility of campaign-style distribution to reach first-time testers, men and adolescents**.** However, we do not know how many remain unaware of HIVST after distribution campaigns, and who these individuals are. Here we look at factors associated with never having heard of HIVST after community-based campaign-style HIVST distribution in rural Zimbabwe between September 2016 and July 2017.

**Methods:**

Analysis of representative population-based trial survey data collected from 7146 individuals following community-based HIVST distribution to households was conducted. Factors associated with having never heard of HIVST were determined using multivariable mixed-effects logistic regression adjusted for clustered design.

**Results:**

Among survey participants, 1308 (18.3%) self-reported having never heard of HIVST. Individuals who were between 20 and 60 years old {20–29 years: [aOR = 0.74, 95% CI (0.58–0.95)], 30–39 years: [aOR = 0.56, 95% CI (0.42–0.74)], 40–49 years: [aOR = 0.50, 95% CI (0.36–0.68)], 50–59 years [aOR = 0.58, 95% CI (0.42–0.82)]}, who had attained at least ordinary level education [aOR = 0.51, 95% CI (0.34–0.76)], and who had an HIV test before [aOR = 0.30, 95% CI (0.25–0.37)] were less likely to have never heard of HIVST compared with individuals who were between 16 and 19 years old, who had a lower educational level and who had never tested for HIV before, respectively. In addition, non-household heads or household head representatives [aOR = 1.21, 95% CI (1.01–1.45)] were more likely to report never having heard of HIVST compared to household head and representatives.

**Conclusions:**

Around one fifth of survey participants remain unaware of HIVST even after an intensive community-based door-to-door HIVST distribution. Of note, those least likely to have heard of self-testing were younger, less educated and less likely to have tested previously. Household heads appear to play an important role in granting or denying access to self-testing to other household members during door-to-door distribution. Differentiated distribution models are needed to ensure access to all.

*Trial registration* PACTR, PACTR201607001701788. Registered 29 June 2016, https://pactr.samrc.ac.za/ PACTR201607001701788

**Supplementary Information:**

The online version contains supplementary material available at 10.1186/s12879-022-07027-9.

## Background

Globally, 19% of people living with human immunodeficiency virus (HIV) are undiagnosed [1], with men, young people (i.e., 15–24 years old), rural and key populations least likely being aware of their status [2]. Innovations to close the gaps in testing coverage and reach underserved are urgently required [3]. Investing in additional, innovative HIV testing strategies such as HIV self-testing (HIVST), where individuals take their own test and interpret the result, may both increase testing coverage and decrease inequities in access.

In 2015, Unitaid invested in a large-scale implementation project, called the Self-Testing AfRica (STAR) initiative. The focus was on generating extensive experience and evidence to scale up access to HIVST across Sub-Saharan Africa. Since then, HIVST acceptability, feasibility and scalability has been demonstrated across many delivery models in different Eastern and Southern African countries [4–9] allowing policy makers to select the most appropriate HIVST delivery models for their context to complement their existing national HIV testing strategies [10]. Community-based distribution can occur during campaigns, at events, through mobile outreach or door-to-door [11]. Previous studies have demonstrated the effectiveness of community-led HIVST campaigns, community-based secondary or peer-led distribution in increasing HIV testing in underserved subgroups [12–14]. Community-based door-to-door distribution has been shown to both increase testing coverage among first-time testers, men and adolescents as well as linkage to antiretroviral therapy (ART) [15]. We do not clearly understand who is left out by door-to-door distribution, including what proportion remain unaware of HIVST after distribution campaigns, and who these individuals are. The aim here is to describe those who self-reported having never heard of HIVST despite living in a community receiving intensive door-to-door community-based HIVST distribution to inform future HIVST delivery strategies. This information is essential to optimize coverage of HIVST and to close the gaps in HIV testing.

## Methods

Secondary analysis was undertaken on a subset of the population-based survey data collected as part of a cluster-randomized trial of supply-side financial incentives to increase uptake of HIVST and linkage to post-test services nested within a time-trend analysis of linkage to care. The study has been published elsewhere (PACTR201607001701788) [16].

In brief, trained community-based distributors delivered HIVST kits through door-to-door distribution to all households in 38 rural Zimbabwean communities between September 2016 and July 2017. Distribution was carried out over 19 days (range 19–25 days) per community. Six to eight weeks later, after completion of HIVST distribution, a population-based survey was conducted in four randomly selected National Census Office Enumeration Areas (EA) per community. Within each EA, Open Data kit was used to randomly select 50% of the households for survey participation, with the aim of recruiting 200 adults per community. Only household members older than or equal to 16 years old were eligible for survey completion, after written informed consent. The survey questionnaire was self-administered on electronic tablets, using Audio Computer Assisted Self Interview (ACASI) and included socio-demographic details, history of HIV testing and ART uptake; access to, use and results of self-testing; and uptake of any health services including confirmatory testing and ART following kit distribution (Additional file 1).

The outcome of interest for this analysis was “ever having heard of HIVST”. The variable was defined from the question “Have you heard about HIV self-testing as a method for testing for HIV?”. Guided by the definition for HIV self-testing: “HIV self-testing is a process whereby a person who wants to know his or her HIV status collects a specimen, performs a test, and interprets the test result in private”. A binary yes or no response was captured. As the research objective of the analysis is focussing on the study population who never heard of HIVST, the reference category for analysis is those who have ever heard of HIVST.

Based on the questions asked in the population-based survey, the following socio-demographic and socio-economic characteristics were considered for the analysis: age, sex, marital status, highest level of education, able to read newspaper or letter, religion, tribe, occupation, regular salary, perception of general health, having lived in the community for the last 12 months, ever tested for HIV and whether one was a head of the household. A household head was defined as the person who is regarded as the custodian of the family and is deferred to for the important decisions of the household. A household representative is the person in charge of the household when the household head is absent. Common mental disorders were measured using the Shona Symptom Questionnaire (SSQ), an indigenous 14-item measure in the Shona language, developed in Zimbabwe, with a cut-off point of 9/14 [17]. All continuous variables were changed into categorical variables to understand how the outcome was distributed among sub-populations. Categorical variables were constructed based on the answer options provided in the population-based survey.

The analysis was conducted in STATA 15.1 (Stata Corporation, College Station, TX, USA). Descriptive analyses describe the characteristics of the survey participants. Factors associated with never having heard of HIVST were determined using multivariable mixed-effects logistic regression of individual-level data with adjustment for random effects to account for the clustered unit of randomisation, i.e., household and community level. To select the final minimum adequate model, a backward stepwise selection reduction was applied using the likelihood-ratio test to assess the goodness of fit of two competing statistical models. All independent variables were included in the model except for ‘regular salary’ and ‘able to read a newspaper or letter’. These latter variables were excluded as they are interrelated with the variables ‘occupation’ and ‘highest level of education’.

## Results

### Characteristics of survey participants

The population-based survey included 7146 people from 3813 households, with a response rate of 83.4%. Not being at home (n = 1091, 12.7%) was main reason for non-response. Almost three quarters of the households (n = 2769, 72.6%) received an HIVST kit during the distribution campaign. The sample included 2767 men (38.7%) and 4379 women (61.3%). About 42% (n = 3001) of the participants were between 20 and 39 years old. Almost 60% (n = 4240) were married. The predominant religion was Apostolic (34.3%, n = 2450). Most participants were Shona (69.3%, n = 4949). About 46% of the participants (n = 3333) were not a household head or a household head representative. Almost half of the survey population (43.3%, n = 3092) had no education or only primary education. Being a subsistence farmer (64.7%, n = 4620) was the main occupation with 16.1% (n = 1153) earning a regular salary. More than half of the participants perceived their general health to be very good (25.9%, n = 1852) or good (37.9%, n = 2713) and 45.1% (n = 3221) of the participants had a SSQ score above 9 points suggesting they were at risk of common mental disorders. Although 6335 participants (88.7%) reported ever having tested for HIV in the past, 18.3% (n = 1308) self-reported that they had never having heard of HIVST.

### Factors associated with never having heard of HIVST

The multivariable mixed-effects analysis shows that participants between 20 and 60 years old are less likely to have never heard of HIVST {20–29 years: [aOR = 0.74, 95% CI (0.58–0.95)], 30–39 years: [aOR = 0.56, 95% CI (0.42–0.74)], 40–49 years: [aOR = 0.50, 95% CI (0.36–0.68)], 50–59 years [aOR = 0.58, 95% CI (0.42–0.82)]} compared to participants between 16 and 19 years old (Table 1). Individuals who have been living in the area for the last 12 months [aOR = 0.48, 95% CI (0.36–0.63)], who had an HIV test before [aOR = 0.30, 95% CI (0.25–0.37)] and who have attained at least ordinary level education [aOR = 0.51, 95% CI (0.34–0.76)] are less likely to have never heard of HIVST than individuals who had not lived in the area over the last 12 months, who had never tested for HIV before and who have a lower educational level.

**Table 1:** Results multivariable mixed effect logistic regression adjusted for clustering on household and community level

Individual characteristics	Ever heard of HIVST N = 5838 (Row %)	Never heard of HIVST N = 1308 (Row %)	OR*	95% CI	P-value	aOR^$^	95% CI	P-value
Age in groups								
16–19 years	820 (74.3%)	284 (25.7%)	–	–	< 0.001	–	–	< 0.001
20–29 years	1300 (82.4%)	277 (17.6%)	0.57	0.46–0.71		0.74	0.58–0.95	
30–39 years	1239 (87.0%)	185 (13.0%)	0.38	0.30–0.49		0.56	0.42–0.74	
40–49 years	929 (87.6%)	131 (12.4%)	0.35	0.27–0.45		0.50	0.36–0.68	
50–59 years	662 (84.0%)	126 (16.0%)	0.49	0.37–0.64		0.58	0.42–0.82	
60 years and older	867 (75.1%)	288 (24.9%)	0.94	0.75–1.17		0.99	0.72–1.36	
Constant	–	–	0.27	0.21–0.34	< 0.001	–	–	–
Sex								
Male	2213 (79.9%)	554 (20.1%)	–	–	< 0.001	–	–	–
Female	3625 (82.8%)	754 (17.2%)	0.80	0.70–0.92		–	–	–
Constant	–	–	0.18	0.15–0.22	< 0.001	–	–	–
Marital status								
Married	3585 (84.6%)	655 (15.4%)	–	–	< 0.001	–	–	–
Never married	1227 (75.9%)	390 (24.1%)	1.92	1.63–2.27		–	–	–
Widowed/separated/divorced	1026 (79.6%)	263 (20.4%)	1.50	1.24–1.82		–	–	–
Constant	–	–	0.12	0.10–0.15	< 0.001	–	–	–
Highest level of education								
None/primary	2412 (78.1%)	680 (21.9%)	–	–	< 0.001	–	–	< 0.001
Some secondary	1556 (81.4%)	355 (18.6%)	0.82	0.69–0.96		0.84	0.70–1.02	
O levels complete^µ^	1571 (87.4%)	227 (12.6%)	0.50	0.42–0.61		0.58	0.46–0.72	
A levels and above^£^	299 (86.7%)	46 (13.3%)	0.54	0.37–0.78		0.51	0.34–0.76	
Constant	–	–	0.21	0.17–0.25	< 0.001	–	–	–
Able to read newspaper or letter								
No	866 (72.8%)	323 (27.2%)	–	–	< 0.001	–	–	–
Yes	4972 (83.5%)	985 (16.5%)	0.50	0.42–0.59		–	–	–
Constant	–	–	0.28	0.23–0.35	< 0.001	–	–	–
Religion								
Apostolic	2005 (81.8%)	445 (18.2%)	–	–	0.245	–	–	–
Other Christian denomination	1863 (83.1%)	380 (16.9%)	0.99	0.83–1.19		–	–	–
Other, including no religion	1970 (80.3%)	483 (19.7%)	1.14	0.95–1.35		–	–	–
Constant	–	–	0.15	0.12–0.19	< 0.001	–	–	–
Tribe								
Shona	4142 (83.7%)	807 (16.3%)	–	–	< 0.001	–	–	0.0060
Ndebele	925 (78.5%)	254 (21.5%)	1.43	1.14–1.79		1.28	1.02–1.61	
Kalanga	489 (75.2%)	161 (24.8%)	1.76	1.36–2.29		1.58	1.21–2.07	
Tonga	23 (82.1%)	5 (17.9%)	1.21	0.39–3.70		0.93	0.29–3.03	
Other	250 (76.5%)	77 (23.5%)	1.66	1.20–2.31		1.47	1.04–2.07	
I don’t wish to answer	9 (69.2%)	4 (30.8%)	2.70	0.67–10.94		2.56	0.61–10.73	
Constant	–	–	0.14	0.12–0.17	< 0.001	–	–	–
Occupation								
Student	531 (75.9%)	168 (24.1%)	–	–	< 0.001	–	–	
Subsistence farmer	3836 (83.1%)	784 (16.9%)	0.57	0.46–0.72		0.83	0.62–1.10	
Self-employed	1045 (80.2%)	258 (19.8%)	0.70	0.54–0.92		1.08	0.79–1.46	0.0131
Formal employment	426 (81.3%)	98 (18.7%)	0.68	0.49–0.95		1.17	0.81–1.70	
Constant	–	–	0.25	0.19–0.32	< 0.001	–	–	–
Regular salary								
No	4909 (81.9%)	1084 (18.1%)	-	-	0.327	–	–	–
Yes	929 (80.6%)	224 (19.4%)	1.10	0.91–1.33		–	–	–
Constant	–	–	0.16	0.13–0.19	< 0.001	–	–	–
Perception general health								
Very good	1534 (82.8%)	318 (17.2%)	–	–	0.027	–	–	0.0165
Good	2247 (82.8%)	466 (17.2%)	0.96	0.80–1.15		1.04	0.85–1.25	
Fair	1184 (80.9%)	278 (19.1%)	1.02	0.83–1.26		1.10	0.88–1.38	
Poor	797 (78.8%)	214 (21.2%)	1.20	0.96–1.51		1.30	1.01–1.67	
Don’t want to answer	76 (70.4%)	32 (29.6%)	1.93	1.16–3.22		2.15	1.27–3.65	
Constant	–	–	0.16	0.13–0.19	< 0.001	–	–	–
Living here last 12 months								
No	292 (70.7%)	121 (29.3%)	–	–	< 0.001	–	–	< 0.001
Yes	5546 (82.4%)	1187 (17.6%)	0.44	0.33–0.57		0.48	0.36–0.63	
Constant	–	–	0.34	0.25–0.46	< 0.001	–	–	–
SSQ ≥ 9								
No	490 (60.4%)	732 (39.6%)	–	–	0.262	–	–	–
Yes	5348 (84.4%)	576 (15.6%)	0.92	0.80–1.06		–	–	–
Constant	–	–	0.16	0.14–0.20	< 0.001	–	–	–
Ever tested for HIV								
No	3193 (81.3%)	321 (18.7%)	–	–	< 0.001	–	–	< 0.001
Yes	2645 (82.1%)	987 (17.9%)	0.24	0.20–0.30		0.30	0.25–0.37	
Constant	–	–	0.55	0.44–0.69	< 0.001	–	–	–
Household head								
Household head	2398 (83.2%)	485 (16.8%)	–	–	0.0008	–	–	0.1002
Household head representative	777 (83.6%)	153 (16.4%)	0.93	0.74–1.17		1.07	0.83–1.37	
Not head/represent	2663 (79.9%)	670 (20.1%)	1.27	1.10–1.47		1.21	1.01–1.45	
Constant	–	–	0.14	0.12–0.18	< 0.001	–	–	–
Constant multivariable model	–	–	–	–	–	1.30	0.84–2.03	0.245

*OR: odds ratio, bivariable analysis, adjusted for clustering on community and household level, people having heard of HIVST as reference group; $ = aOR: adjusted odds ratio, multivariable mixed-effect logistic regression, adjusted for clustering on community and household level after backward reduction, people having heard of HIVST as reference group; µ = Ordinary (O) Level—basic level of the General Certificate of Education completed in the third/fourth years secondary school, a subject-based qualification; £ = Advanced (A) Level—advanced level of the General Certificate of Education completed in the fifth/sixth years of secondary school, a subject-based qualification

Non-Shona people, except for the Tonga people, had a statistically significant increased odds of not having heard about HIVST compared to Shona people Ndebele: [aOR = 1.28, 95% CI (1.02–1.61)], Kalanga: [aOR = 1.58, 95% CI (1.21–2.07)], Other: [aOR = 1.47, 95% CI (1.04–2.07)]. Individuals who perceive their health to be poor [aOR = 1.30, 95% CI (1.01–1.67)], or who did not answer the general health perception question [aOR = 2.15, 95% CI (1.27–3.65)] have increased odds of never having heard of HIVST compared with those who perceive their general health to be good. Individuals who are not household heads or household head representatives have increased odds of never having heard of HIVST compared to household heads or household head representatives [aOR = 1.21, 95% CI (1.01–1.45)].

No significant association was found between having never heard of HIVST among individuals with a formal employment compared to those in other employment categories [aOR = 1.17 95% CI (0.81–1.70)].

## Discussion

This study provides insight into factors associated with never having heard of HIVST after intensive community-based campaign-style HIVST distribution in rural Zimbabwe. Among survey participants, nearly one fifth self-reported having never heard of HIVST. Individuals who were between 16 and 19 years old, who had no formal education or had only attended primary school and who had never tested before for HIV were more likely to have never heard of HIVST. In addition, those who were not household heads or household head representatives were more likely to have never heard of HIVST.

Understanding who is missed by door-to-door community-based test distribution will be helpful for designing future HIVST distribution models [18]. Zimbabwe was an early adopter of HIVST. At a time when small HIVST pilot studies were being implemented [19], the Zimbabwean 2015–2016 Demographic and Health Survey (DHS) data showed a population-level awareness for HIVST of only 14.5% [20]. Comparing this low percentage with the 81.7% found in this study, shows the positive impact of large-scale implementation studies, such as the STAR Initiative on awareness. As there is a constant evolution in exposure and presence of HIVST overtime, it remains important to examine awareness trends in future national surveys such as DHS.

Similar conclusions in terms of those aware of HIVST were found in the 2015–2016 DHS. Awareness was lower among respondents who were younger (below 20 years) and with lower levels of education (primary education or less) [20]. This is in line with other studies which found that HIV testing and knowledge of HIV status increases with age and educational level [21–23]. Possible explanations may be linked to general trends in HIV testing. Younger people and those less educated are less knowledgeable about HIV, and thus may know less about different HIV testing options, including self-testing [24, 25]. Despite the high HIVST awareness achieved by our community-based distribution model, culminating in high testing rates among individuals who might not otherwise test (i.e., men, young people, those testing for the first time), some still remained unaware of their HIV status [26–29]. Complementing community-based distribution with other HIVST distribution models such as those through health facilities and youth clinics, by sexual partners or secondary distribution and workplace programmes will be needed to reach those remaining unaware [30–36].

Non-household heads were more likely to not have heard of HIVST. As was reported in qualitative studies that were done alongside this research (data not reported here), this may be because when distributors approach households, they are culturally expected to first approach the head of household, who may not invite the rest of the household to the discussion [37]. Future efforts should ensure that everyone in a household is invited to discussions, with platforms created for separate discussions as appropriate (e.g., young people would like HIVST discussions to be held separately from their parents/guardians) [37]. A community-based program on HIVST in rural Malawi found that HIVST was more prevalent among individuals who shared a household with someone who reported HIVST [29]. Understanding the role of household dynamics on facilitating community-based distribution activities within the household should be further investigated. Alongside, alternative distribution methods should be implemented for those who are uncomfortable accepting and performing an HIV self-test in the presence of other household members, for example, through youth centres or peer to peer distribution for young people [37–41] Furthermore, appropriate promotion tools such as mobile platforms and social media technology should be used to increase HIVST awareness among young people [39].

The population-based survey outcomes were based on self-reporting, which may have been subject to social desirability bias. To minimize this bias, ACASI was used [42]. Despite this, this study provides new insights into characteristics of individuals who remain unaware of HIVST following community-based HIVST kit distribution and confirms the presence of ongoing barriers to HIV testing. Lastly, our results are specific for community-based door-to-door distribution of HIVST kits. Other distribution models or a different intervention design might have affected awareness of HIVST differently.

## Conclusions

Around one fifth of survey participants remain unaware of HIVST even after an intensive community-based door-to-door HIVST distribution. Of note, those least likely to have heard of self-testing were younger, less educated and less likely to have tested previously. Household heads appear to play an important role in granting or denying access to self-testing to other household members during door-to-door distribution. Differentiated distribution models are needed to ensure access to all.

## Supplementary Information

**Additional file 1.** Household questionnaire Zimbabwe.

## Data Availability

The datasets used and/or analysed during the current study are available from the corresponding author on reasonable request.

## Abbreviations

ACASI: Audio computer assisted self interview
ART: Antiretroviral therapy
DHS: Demographic and health survey
EA: Enumeration area
HIV: Human immunodeficiency virus
HIVST: HIV self-testing
SSQ: Shona Symptom Questionnaire
STAR: Self-Test Africa
